# Diagnosis and Management of Aggressive/Refractory Growth Hormone-Secreting Pituitary Neuroendocrine Tumors

**DOI:** 10.1155/2024/5085905

**Published:** 2024-08-26

**Authors:** Xiaojuan Zhang, Yu Chen, Yerong Yu, Jianwei Li

**Affiliations:** Department of Endocrinology and Metabolism West China Hospital of Sichuan University, Chengdu, China

## Abstract

The majority of acromegaly and gigantism are caused by growth hormone-secreting pituitary neuroendocrine tumors (PitNETs). Most cases can be cured or controlled by surgery, medical therapy, and/or radiotherapy. However, a few of these tumors are resistant to traditional therapy and always have a poor prognosis. The title aggressive/refractory is used to differentiate them from pituitary carcinomas. To date, there is no definitive conclusion on how to diagnose aggressive/refractory growth hormone-secreting PitNETs, which may have slowed the process of exploring new therapeutical strategies. We summarized the literature described diagnosis and treatment of the disease. Potential disease markers and prospective therapies were also included.

## 1. Introduction

Pituitary neuroendocrine tumors (PitNETs) are a group of neoplasms derived from adenohypophyseal cells [1]. Most PitNETs are benign adenomas that can be cured or controlled by surgery, medical therapy, or radiotherapy. Very rare (0.1%–0.2%) PitNETs are malignant tumors defined as pituitary carcinomas that exhibit with craniospinal dissemination and/or systemic metastases [2]. According to 2022 WHO classification of pituitary tumors, the term pituitary carcinoma is suggested to be replaced by metastatic PitNETs [3]. About 0.5%–10% of PitNETs are aggressive/refractory PitNETs, and the variation is largely due to different diagnostic standards [2, 4]. Among them, nearly 4.5% to 31% are growth hormone (GH)-secreting aggressive/refractory PitNETs [5, 6]. Hypersecretion of GH leads to excess insulin-like growth factor-1 (IGF-1) and results in acromegaly in adults and gigantism in children and adolescents. The handling of aggressive/refractory acromegaly and gigantism is of great challenge. We searched in PubMed with keywords included but not restricted to the following: refractory pituitary adenoma, aggressive pituitary adenoma, growth hormone, acromegaly, gigantism, and pituitary neuroendocrine tumors. The relevant literature was reviewed to summarize current opinions on the definition, potential markers, diagnosis, and management of aggressive/refractory GH-secreting PitNETs.

## 2. Definition and Potential Markers of Aggressive/Refractory GH-Secreting PitNETs

**Figure 1:**
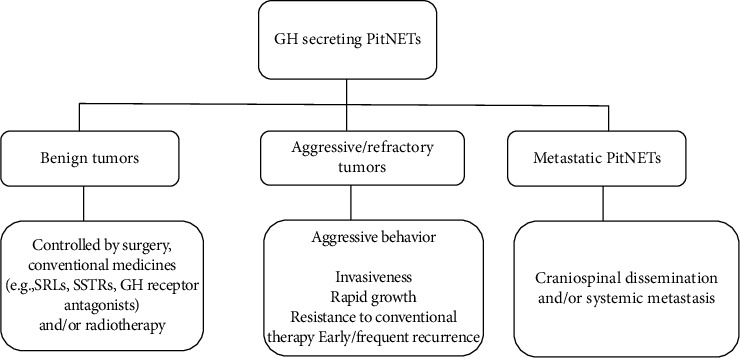
Clinical features of GH-secreting PitNETs. GH, growth hormone; PitNETs, pituitary neuroendocrine tumors; SRLs, somatostatin receptor ligands; SSTRs, somatostatin receptors.

**Figure 2:**
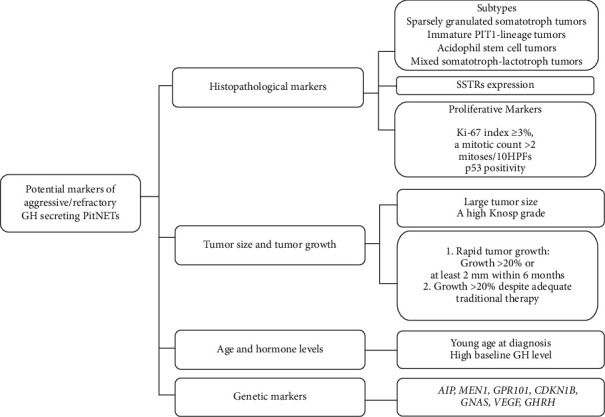
Potential markers of aggressive/refractory GH-secreting PitNETs. GH, growth hormone; PitNETs, pituitary neuroendocrine tumors; SSTRs, somatostatin receptors.

The definition of aggressive/refractory PitNETs as well as aggressive/refractory GH-secreting PitNETs has not been clearly demonstrated. According to previous investigations, the characteristics of aggressiveness/refractoriness of PitNETs generally include invasiveness to surrounding structures or sinuses, rapid growth, resistance to conventional therapy, and early/frequent recurrence (shown in Figure 1) [7–9]. Since aggressive/refractory GH-secreting PitNETs always indicate a poor prognosis in patients with acromegaly and gigantism, early identification of cases with disease markers is of great value for patient care. In recent decades, many efforts have been made to explore predictive and prognostic markers and factors that display the aggressiveness/refractories of GH-secreting PitNETs. Unfortunately, up to date, no definitive conclusion has been made on which markers alone or in combination are validated to be used for identifying aggressive/refractory GH-secreting PitNETs and predicting their prognosis. We summarized some markers that have been widely discussed in previous studies (shown in Figure 2).

### 2.1. Histopathological Markers

#### 2.1.1. Subtypes of GH-Secreting PitNETs That Show Aggressiveness and Refractoriness

In 2022, the WHO formally chose the term PitNETs to replace pituitary adenomas [3]. Additionally, significant progress has been made in the classification of pituitary tumors, which are now classified according to cell lineage as determined by expression of transcription factors, hormones, and other biomarkers [3]. PIT1, TPIT, and SF1 are three major pituitary transcription factors. According to the WHO 2022 classification, PitNETs are divided into four types: PIT1-lineage PitNETs (mainly consist of somatotroph tumors, thyrotroph tumors, and lactotroph tumors), TPIT-lineage PitNETs (corticotroph tumors), SF1-lineage PitNETs (gonadotroph tumors), and PitNETs with no distinct cell lineage. GH-secreting PitNETs are derived from cells of PIT1 lineage. Except for somatotroph tumors, which is the major source of GH excess, PIT-lineage-derived mammosomatotroph tumors, mature plurihormonal PIT1-lineage tumors, immature PIT1-lineage tumors, acidophil stem cell tumors, and mixed somatotroph-lactotroph tumors also preserve the ability to secrete excess GH.

**Figure 3:**
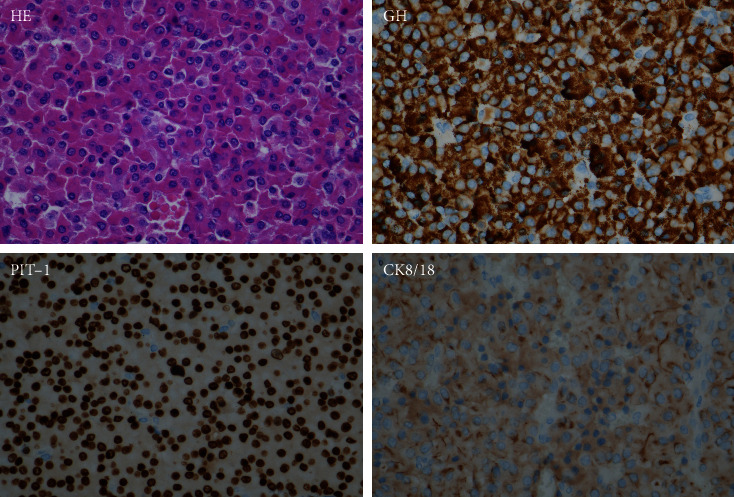
Histology of densely granulated somatotroph tumor. These tumors are composed of large cells with acidophilic cytoplasm, diffuse strong cytoplasmic reactivity for GH, nuclear positivity for PIT1, and diffusely distributed cytokeratin using the CK8/18 stain. HE, hematoxylin-eosin staining; GH, growth hormone; PIT-1, pituitary-specific transcription factor-1; CK8/18, cytokeratin 8/18.

**Figure 4:**
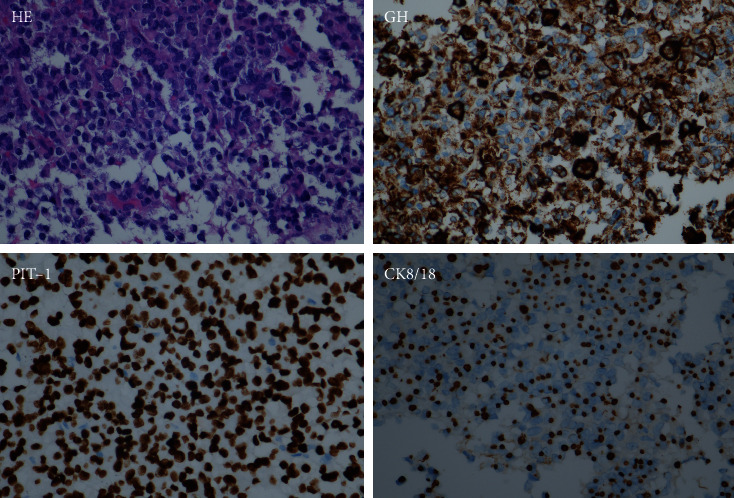
Histology of sparsely granulated somatotroph tumor. These tumors are composed of large cells with chromophobic cytoplasms that harbor keratin aggresomes known as fibrous bodies and are positive for PIT-1. They have scant-variable cytoplasmic reactivity for GH and dots staining for CK8/18 in fibrous bodies. HE, hematoxylin-eosin staining; GH, growth hormone; PIT-1, pituitary-specific transcription factor-1; CK8/18, cytokeratin 8/18.

**Figure 5:**
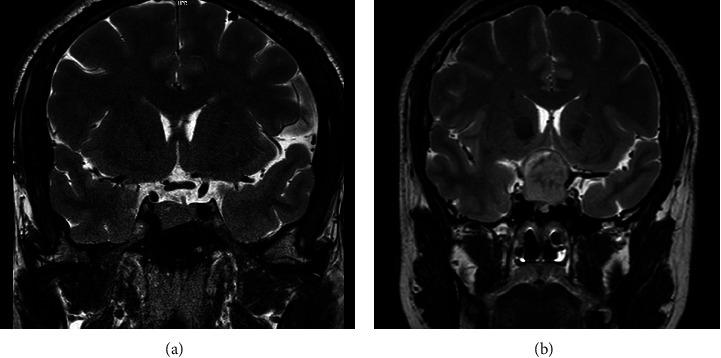
MRIs of GH-secreting PitNETs. (a) T2-weighted MRI of a patient with densely granulated somatotroph tumor. Tumor size 1.7 cm × 1.6 cm × 1.1 cm (arrow). (b) T2-weighted MRI of a patient with sparsely granulated somatotroph tumor. Tumor size 2.5 cm × 2.6 cm × 2.7 cm (arrow). MRI, magnetic resonance image; GH, growth hormone; PitNETs, pituitary neuroendocrine tumors.

Somatotroph tumors have two subtypes, namely, densely granulated somatotroph tumors and sparsely granulated somatotroph tumors. The former contributes to half of the excess GH, and the latter is also a common cause of GH hypersecretion [10]. The two subtypes of somatotroph tumors are differentiated by the density of GH-containing secretory granules and the cytoplasmic distribution of immunoreactive cytokeratin (Figures 3 and 4). Densely granulated somatotroph tumors typically show hypointense on T2-weighted magnetic resonance image (MRI), while sparsely granulated somatotroph tumors are more frequently present with T2 hyperintensity on MRI [11] (Figure 5). Plenty of evidence has shown that sparsely granulated somatotroph tumors exhibit invasive and/or aggressive behavior [10, 12, 13]. The other types of GH-secreting PitNETs that present with invasive and/or aggressive behavior include tumors of immature PIT1 lineage, acidophil stem cell tumors, and mixed somatotroph-lactotroph tumors [11]. The determination of the subtypes of GH-secreting PitNETs has been described in detail by Asa et al. [11].

#### 2.1.2. Somatostatin Receptor Expression

Subtypes of somatostatin receptors (SSTRs), mainly SSTR2 and SSTR5, mediate most of the effects of the clinically available somatostatin receptor ligands (SRLs). Resistance to SRLs has been reported to be associated with different expression levels of SSTRs in GH-secreting PitNETs, though the results are controversial in different studies [13]. Generally speaking, biochemical and tumoral responses to first-generation SRLs, octreotide, positively correlate with SSTR2 expression, while negatively correlate with SSTR5 expression, respectively [13]. Mixed results are found in studies evaluating the responses to second-generation SRLs, pasireotide [13], while for the evaluation of tumor invasion, Zhang et al. show in their study that it is negatively correlated with SSTR2 expression [14].

#### 2.1.3. Proliferative Markers

Unlike other tumors, typical morphological indicators of tumor aggression, for example, pleomorphism and nuclear atypia, do not correlate well with the malignant potential of PitNETs [15, 16]. Proliferative markers, e.g., Ki-67 index, p53 positivity, and increased mitotic activity, are frequently being used as predictors of the prognosis of PitNETs [17]. Although the cutoff value varies significantly, a Ki-67 index ≥3%, a mitotic count >2 mitoses/10HPFs, and p53 positivity were primarily observed and discussed [15, 18, 19] in aggressive/refractory PitNETs. In a cohort of 61 patients with acromegaly, tumors with a Ki-67 value >3% exhibited with more aggressive behavior, more manifestations of acromegaly, higher GH level, and higher recurrence compared with lower Ki-67 value (<3%) [20]. The largest survey conducted in patients with aggressive pituitary tumors (*n* = 121) and carcinomas (*n* = 50) so far reported that a Ki-67 index ≥3% was observed in the majority of patients and the value increased over time in patients who had repeat surgery [21]. Although Ki67 index is not recommended by WHO to grade various PitNET tumor types, as there are other biomarkers of aggressive behavior of different PitNETs [3], undeniably, proliferative markers play a role in predicting the aggressiveness and prognosis of GH-secreting PitNETs [22].

### 2.2. Tumor Size and Tumor Growth

In 2018, ESE published the data from 165 patients with aggressive pituitary tumors and carcinomas, resulting in the first guideline for the management of the disease [23]. Later in 2022, the survey was extended in sample size. A total of 171 patients were included in the second survey, and the vast majority of them were reported to have macroadenomas (*n* = 125/168) and giant tumors (*n* = 37) [21]. The most common tumors were nonfunctioning prolactin-positive or ACTH tumors. GH-secreting PitNETs were not common in that survey [23]. Those tumors with extrasellar extension and invasion of adjacent structures have been shown to be more aggressive [22]. In patients with GH-secreting PitNETs who underwent transsphenoidal surgery for tumor resection, the Knosp grade 3-4 classification was significantly associated with nonremission outcomes [24]. Therefore, large tumor size and high Knosp grade are prognostic factors of GH-secreting PitNETs.

The revised Response Evaluation Criteria in Solid Tumors (RECIST) is frequently used to evaluate relevant tumor growth in PitNETs [15, 25]. Growth >20% and at least 2 mm in 6 months are considered as rapid tumor growth. Growth >20% despite adequate surgery, medical treatment, and radiotherapy are defined as clinically relevant tumor growth despite optimal conventional therapy. Both indicate the aggressiveness of the disease.

### 2.3. Age and Hormone Levels

Younger patients with acromegaly and gigantism have been shown to have a higher GH level and larger tumors compared to older patients [26–28]. Therefore, the young age at diagnosis, the high baseline GH level and the large tumor size together indicate a more invasive and aggressive phenotype [22]. Acromegaly and gigantism patients diagnosed at young age are recommended by ESE to undergo genetic tests to verify gene mutations [17], which will be described in details in the next section.

### 2.4. Genetic Markers

The genetic markers of GH-secreting PitNETs that predict disease behavior have been extensively reviewed by Hernández-Ramírez in 2020 [13] and 2024 [29]. About 5–7% of PitNETs occur in a family setting, and the proportion of familial cases is higher in young patients [24]. Among them, most cases are familial isolated pituitary adenoma [30] and multiple endocrine neoplasia type 1 (MEN1) [24]. In young-onset acromegaly and gigantism, about 20% are due to a germline defect [31, 32]. The genetic cause of FIPA remains largely unknown. Approximately one fifth are caused by germline variants of loss of function in the aryl hydrocarbon receptor-interacting protein gene [33, 34]. Most patients with AIP mutation positive for GH-secreting PitNETs have macroadenoma, mostly possess sparsely granulated type, and are resistant to first-generation SRLs and dopamine agonists, which always require multimodal treatment [30, 35]. Most MEN1 patients have germline loss-of-function mutations in the *MEN1* gene [36]. More than a third of MEN1 patients develop PitNETs and 9% are associated clinically with somatotropinomas [37]. These tumors are significantly larger, more invasive, and have a poor response to surgical and/or medical treatment [37]. X-linked acrogigantism, a specific type of gigantism that occurs in early infancy, compromises the features of both acromegaly and gigantism. It is known to be the result of Xq26.3 microduplications encompassing the GPR101 gene. Patients with X-linked acrogigantism have a poor response to first-generation SRLs and are also resistant to surgery or radiotherapy as well. Likewise, acromegaly and gigantism that caused by other genetic defects, such as *CDKN1B* and *GNAS*, always harbor the features of aggressive/refractory GH-secreting PitNETs [13]. The ESE guideline suggested germline genetic testing based on young age at presentation or family history of pituitary or endocrine neoplasia [17] in patients diagnosed with aggressive pituitary tumors [17]. Despite germline gene defects, dysregulated expression of specific genes, for example, *VEGF* and *GHRH*, is associated with invasiveness or neoplastic progression and clinical aggressiveness of GH-secreting PitNETs [38, 39].

## 3. Diagnosis of Aggressive/Refractory GH-Secreting PitNETs

Guidelines for the diagnosis and management of GH-secreting PitNETs have been well-established and regularly updated [39–42]. On the contrary, there is no consensus on how to diagnose aggressive/refractory PitNETs as well as aggressive/refractory GH-secreting PitNETs. Different diagnostic criteria are used by different researchers and clinicians. In 2004 WHO Classification of Pituitary Tumors, Ki-67 ≥ 3% was applied to define atypical tumor. However, this item was removed from 2017 version, as it could not be well validated. High-risk pituitary adenomas with clinical aggressive behavior were mentioned in the 2017 WHO Classification of Pituitary Tumors. Tumors with radiological invasion, rapid growth, and a Ki-67 index>3% are considered as high-risk pituitary adenomas. In addition, some PitNET subtypes are recognized as high-risk tumors, including sparsely granulated somatotroph adenoma and plurihormonal Pit-1-positive adenoma that are associated with acromegaly and gigantism. In 2018, ESE guideline recommended diagnosis of an aggressive pituitary tumor be considered in patients with a radiologically invasion, rapid tumor growth, or clinically relevant tumor growth despite optimal standard therapies. However, no specific definition was given to determine tumor invasiveness, rapid growth rate, and clinically relevant tumor growth. Recently, Raverot et al. summarized previous diagnostic criteria of aggressive pituitary tumor and provided a helpful figure diagram to illustrate tumor growth, assessment of treatment response, and clinically relevant tumor growth [15]. If the tumors cause neuroophthalmological complications, it is directly considered as “clinical relevance.” The authors proposed that the evaluation of the Ki-67 index and mitotic count be included. When the Ki-67 index is ≥3%, the immunodetection of p53 is also recommended. Furthermore, they supported the notion that a change in secretory pattern may predict of tumor aggressiveness.

In 2016, a novel concept of “refractory pituitary adenoma” was raised by Dai et al. [18], which is defined as follows: (1) tumor infiltrates adjacent structures according to imaging and surgical findings, (2) the Ki-67 index is over 3% and growth velocity is more than 2% per month, (3) current treatments fail to control tumor growth and/or hormonal hypersecretion, (4) tumor recurrence occurs within 6 months after surgery. Although this definition is more strict and clearer, it has some drawbacks. For example, it would be difficult to evaluate tumor growth velocity by imaging every month [6], although ideally aggressive tumors should be monitored more frequently. In addition, a tumor growth >20% is considered significant, as recommended by RECIST, since lower percentages of growth are within the measurement error [43]. By adopting the revised RECIST standards, Kasuki and Raverot [25] define and diagnose aggressive pituitary tumor as (1) invasive of the tumor and usually rapid tumor growth (Knosp grades 3–4, invasion of sphenoid sinus (surgery or pathological findings)) and growth >20% and at least 2 mm within 6 months, and (2) clinically relevant tumor growth despite optimal conventional therapy (growth >20% despite adequate surgery, medical treatment, and radiotherapy).

When diagnosing aggressive/refractory GH-secreting PitNETs, referring to previous standards in combination with specific features of GH-secreting PitNETs would be a reasonable approach. In 2017, Donoho et al. [44] proposed to diagnose aggressive acromegaly in their review as (1) rapid tumor growth, age less than 30 years at diagnosis, progressive increase in size despite therapy, and resistance to medical therapy; (2) imaging features, including extrasellar invasiveness and Knosp classification [7, 45]; (3) histopathological features, such as increased Ki-67 labeling index, increased expression of p53, and sparsely granulation. However, the details on how to determine tumor growth rate, progressive increase in size, and the cutoff value of Ki-67 and p53 expression were not given. There is a risk score system proposed by Fernandez-Rodriguez et al. to predict prognosis of GH-secreting PitNETs [22]. Factors that include histology sparsely granulated tumor, MRI T2 intensity, younger age, Ki-67 > 3%, higher baseline levels of GH/IGF-1, tumor volume (T4/T5), and increased tumor size. Factors do not equally impact the prognosis of acromegaly and gigantism patients. Among them, increasing tumor size possesses the highest score of 3, then followed by score 2 factors: T2 magnetic resonance intensity, younger age, Ki-67 > 3%, higher baseline GH/IGF-1 levels, and tumor volume (T4/T5). Sparsely histology granulation has the lowest score of 1. The higher the sum score, the more aggressive the tumor is. Unfortunately, in detail, it is not described how the scores are determined and the cutoff value of the sum score is not provided to make a diagnosis of aggressive/refractory GH-secreting PitNETs. A recent systematic review shows that tumor size and invasiveness are the two main criteria for nonremission of acromegaly due to incomplete resection in more than 70 percent of cases. But this is due more to the difficulties of the surgical technique itself than to the intrinsic characteristics of the tumor [46]. Bianchi et al. recently presented a helpful flow chart for the management of difficult/aggressive GH-secreting PitNETs [47]. In their review, difficult GH adenomas were defined as tumors not cured/controlled after neurosurgical (first-line) and first-generation somatostatin SRL (second-line) therapy. In our opinion, difficult GH adenomas cover more tumors other than refractory/aggressive GH-secreting PitNETs.

Based on the criteria of Dai et al. [17] and other previous publications, we propose in this review the diagnosis of aggressive/refractory GH-secreting PitNETs as (1) GH-secreting PitNETs invade adjacent structures according to imaging and intraoperative findings; (2) the Ki-67 index is over 3%, or sparsely histology granulation with T2 hyperintensity on MRI and age less than 30 years, or plurihormonal PIT-1-positive tumor; (3) tumor growth (volume or diameter) is greater than 20% and/or 2 mm within 6 months; (4) clinically relevant tumor growth (referring specifically to Raverot et al. [15]) despite standard therapies; and (5) tumor recurrence occurs within 6 months after surgery.

## 4. Management of Aggressive/Refractory GH-Secreting PitNETs

### 4.1. Multidisciplinary Team Work

Once GH-secreting PitNET is suspected, a multidisciplinary team (MDT) that includes experts specialized in neuroendocrinology, neurosurgery, neuroradiology, radiation oncology, medical oncology, and neuropathology should be involved in the diagnosis, treatment, and follow-up of the patient, ideally in a Pituitary Tumors Centers of Excellence (PTCOEs) in a single institution [48]. Personalized therapy would be conducted according to clinical guidelines, case-by-case condition, and medications available in different regions [12, 49, 50]. Although the treatment of these cases is of significant challenge, early identification and diagnosis of aggressive/refractory GH-secreting PitNETs is critical, as more aggressive strategies can be used earlier for a probably better prognosis.

### 4.2. Surgery

As transsphenoidal surgery is the first-line therapy for GH-secreting PitNETs [40], most aggressive/refractory cases have been treated with at least one surgery. In 2018, the ESE guideline for the management of aggressive pituitary tumors recommended that surgery be discussed first before other treatment options, as repeated surgery might still have a role to ameliorate local mass effect or to offer control of hormone hypersecretion [17]. In some cases, a more aggressive surgical approach, for example, transcranial mass resection and manipulation within the cavernous sinus [51, 52], could be necessary, which increased surgical complications together with the severity of the disease [53, 54]. Therefore, the ESE guideline recommended that surgeries should be performed by a neurosurgeon with extensive experience in pituitary surgery after MDT evaluation to avoid the risk of major side effects [17].

### 4.3. Conventional Medical Therapy

Conventional medical therapy for GH-secreting PitNETs includes first-/second-generation SRLs, dopamine receptor agonists, and GH receptor antagonists [55]. Most of the GH-secreting PitNETs express somatostatin receptors (SSTRs) and dopamine receptors. Activation of the two signal pathways inhibits GH secretion and tumor growth. SRLs (e.g., fg-SRL octreotide and sg-SRL pasireotide) are commonly used as adjuvant medical therapy for GH-secreting PitNETs if surgery is failed, contraindicated, or rejected by patients [40, 56]. Some patients may need preoperative treatment to help achieve better clinical outcomes after surgery [40, 57]. In this situation, SRLs are the first-line choice, and the dopamine receptor agonist cabergoline could be considered in a case-by-case approach [57]. Stimulation of GH receptors in the liver produces IGF-1. GH receptor antagonists (pegvisomant) inhibit IGF-1 production and are less commonly used as antisecretory drugs for GH-secreting PitNETs, because they may stimulate tumor growth. Combination therapy with SRL and pegvisomant is considered in some patients [42]. A recent consensus gives an algorithm that clearly shows the steps of when different antisecretory drugs being used for the management of GH-secreting pituitary tumors [48]. Some novel therapies, for example, different formulations of current anti-acromegaly drugs and antisense oligonucleotides of GH receptors, are underinvestigation for better effects and/or patient's compliance [58, 59]. Aggressive/refractory GH-secreting PitNETs have been treated with multimodal therapies, including conventional antisecretory medications. Nevertheless, maximally tolerated doses of standard medical therapy are recommended by ESE, in order to control tumor growth, and SRLs are preferable for this purpose [17]. Gallbladder abnormalities and gastrointestinal symptoms are the main side effects of SRL, which are often mild and transient [57].

### 4.4. Radiotherapy

According to the ESE recommendation for functioning pituitary tumors, radiotherapy is suggested in patients with clinically relevant tumor growth despite surgery and standard medical treatment [17]. In cases with clinically relevant invasive tumor remnants with pathological markers (Ki-67 index, mitotic count, p53 positivity) strongly indicating aggressive behavior, adjuvant radiotherapy should be considered. Tumor size, location, prior radiotherapy, doses, and pathology should be evaluated and discussed with an experienced radiation oncologist about different radiotherapeutic options. Conventional fractionated external beam radiotherapy (EBRT) and modern stereotactic radiotherapy techniques, including fractionated stereotactic radiotherapy (FSRT) and stereotactic radiosurgery (SRS) with one single irradiation fraction, are applicable for GH-secreting PitNETs [42, 58]. The efficacy of different techniques is comparable [60, 61], while modern stereotactic techniques may have less adverse effects, especially SRS [62, 63]. As the adjuvant treatment for GH-secreting PitNETs, FSRT and SRS are preferable to EBRT because earlier biochemical remission is achieved [64, 65] and lower rates of hypopituitarism [62] and post-treatment visual changes [63] are reported. Furthermore, FSRT and SRS probably have a lower risk of secondary malignancy [66], although long-term follow-up is required to draw definitive conclusions. Wilson et al. demonstrated in their study that only three of 86 (4%) patients undergoing SRS had a documented increase in tumor size, and none of the patients undergoing FSRT had a documented increase in size following a median follow-up of about 5 years [67]. Meanwhile, target growth hormone levels were met by 14% and 20% in SRS and FSRT groups, respectively. Zheng et al. recently analyzed 33 studies including 2016 patients and found that SRS and FSRT showed comparable efficacy and safety in the management of patients with GH-secreting pituitary adenomas. SRS might be associated with better biochemical remission [68]. However, stereotactic techniques have their own limitations. For example, SRS is limited to tumors within 3 cm of diameter and at least 3 mm away from optic chiasm [17]. FSRT, on the other hand, is divided into multiple sessions, which is not convenient and may not be well-tolerated. For tumors with irregular anatomy and large size, EBRT might be the only option for radiotherapy [17].

### 4.5. Chemotherapy

Given the nature of the poor response of aggressive/refractory PitNETs to traditional therapies, chemotherapy currently represents to be the last effective choice for these patients. The new nomenclature system may allow early use of chemotherapy in PitNETs that are defined as aggressive/refractory tumors, since it does not emphasize the benign characteristic of adenomas.

Temozolomide (TMZ) is the first-line chemotherapy recommended by ESE for aggressive pituitary tumors and pituitary carcinoma, following documented tumor growth, after failure of standard treatment options [17]. It is a second-generation oral alkylating agent that can cross the blood-brain barrier. TMZ leads to DNA methylation at the O^6^ position of guanine, which ultimately results in tumor cell death. It was initially used as a chemotherapy drug for glioblastoma. In 2006, several studies reported patients with pituitary carcinoma treated with TMZ for the first time [69–71]. Since then, hundreds of patients had been reported to have TMZ treatment for pituitary carcinomas and aggressive pituitary tumors, and the efficacy (complete response and partial response) was around 40% to 60% [21, 23, 72–74]. The respondents were shown to have functional hormone-secreting tumors and low expression of O6-methylguanine DNA methyltransferase (MGMT), a DNA-repair enzyme that counteracts DNA damage induced by TMZ [23, 34, 74, 75]. Luo et al. recently published a systematic review and meta-analysis that included 21 studies and 429 patients with aggressive pituitary tumors (*n* = 302) and pituitary carcinomas (*n* = 126) [74]. The results showed that the overall radiological response rate was 41% and the biochemical response rate was 53%. Their data also indicated that the combination of TMZ and radiotherapy significantly increased the overall radiological response rate [74]. Low/intermediate expression of MGMT predicted better response to TMZ. However, it is not always true that MGMT expression predicts TMZ response [17]. According to the 2018 ESE guideline, MGMT positivity was encouraged to be evaluated prior to TMZ treatment. Although patients with high MGMT expression might not respond to TMZ, a trail of TMZ therapy may still be considered [17]. Common chemotherapy side effects are reported in approximately 1/5 of patients with pituitary tumors treated with TMZ, for example, cytopenias, fatigue, and nausea/vomiting [23].

If monotherapy with TMZ fails to control tumor growth, combination chemotherapy is suggested [17]. Limited studies have described the effects of TMZ and other chemotherapy on aggressive/refractory GH-secreting PitNETs. Based on published data, tumors with low levels of MGMT expression showed a better outcome with TMZ treatment [76, 77]. Recently, a group of authors reported that TMZ combined with capecitabine was more effective in controlling aggressive/refractory GH-secreting PitNETs than TMZ alone in patient-derived 3D spheroid *ex vivo* assay [77]. The patient had received five surgeries, various standard medications, and radiotherapy. Histology showed a sparsely granulated somatotrophic tumor with Ki-67 index increased from 3.2% to 22%. All these indicated the aggressiveness and refractoriness of the tumor. According to the ESE recommendation, TMZ could be used as monotherapy for this patient. The patient had a high expression of tumor MGMT. With the prediction of the *ex vivo* data, the authors used the combination therapy of TMZ and capecitabine (CAPTEM) directly. After CAPTEM treatment, tumor size decreased dramatically and GH and IGF-1 levels were restored to normal range [77]. This study suggests that the *ex vivo* assay is helpful in determining the chemotherapy regimen for aggressive/refractory GH-secreting PitNETs.

In another recent study, patients with refractory pituitary adenoma were randomly divided into the CAPTEM group (*n* = 40, including 16 patients with acromegaly) and the TMZ group (*n* = 40, including 17 patients with acromegaly) [78]. The two groups had comparable adverse events, but the CAPTEM group presented with better overall response rate in a one-year follow-up period. The underlying mechanism was not well discussed, and the expression of MGMT of the tumors was not analyzed in this study. The ESE guideline did not recommend a specific regimen for combination therapy with TMZ. More studies are required to evaluate the safety and efficacy of different combination regimens in the treatment of aggressive/refractory GH-secreting PitNETs.

In a prospective study consisting of 35 patients with aggressive pituitary adenomas in a single medical center, 24 patients were identified to be responders to TMZ (including 13 patients with acromegaly) and the average time it takes for them to receive TMZ treatment after diagnosis was 15 months [34]. 11 patients were found to be nonresponders (including four acromegaly patients). The average time before using TMZ after diagnosis was 36 months. The results indicated that early initiation of TMZ after diagnosis of aggressive pituitary adenomas led to better clinical outcomes without severe adverse events in treatment cycles ranging from 3–15.2 cycles and a follow-up period of 35.2–132 months [34]. The safety and efficacy profile reported in this study suggests that the early use of chemotherapy in aggressive/refractory GH-secreting PitNETs might be applicable for better disease management.

### 4.6. Prospective Therapies

Aggressive/refractory PitNETs are very difficult to manage. If standard therapies and TMZ fail to control the disease, no evidence-based treatment is currently available. Therefore, new treatment options are urgently needed to be developed. There are some emerging therapies that are still underinvestigation.

#### 4.6.1. Estrogen and Selective Estrogen Receptor Modulators (SERMs)

The use of estrogen to treat acromegaly by decreasing liver IGF-1 production can date back to 1930s–1940s [79], although the underlined mechanism has not been fully understood. The mild-to-moderate effect, limitation to female patients, and the development of other antisecretory drugs hold up their wider use in acromegaly patients. In recent decades, with the development of selective estrogen receptor modulators (SERMs), the application of estrogen and SERMs for acromegaly has been brought back to stage [80, 81]. A meta-analysis of several observational studies shows that estrogen therapy produces the greatest reduction in IGF-1 levels in women, and SERMs treatment to a lesser degree [82], particularly in those who do not respond to conventional therapy. Dimaraki et al. demonstrated that raloxifene, a SERM, significantly reduced IGF-1 in men with active acromegaly despite traditional treatments [83]. Clomiphene citrate, another type of SERM, is effective in treating of male acromegaly not controlled by conventional therapies [84]. Therefore, estrogen and SERMs can be used for aggressive/refractory GH-secreting PitNETs. Recently, a prospective study observed normalization of IGF-1 in 3 of 8 patients and reduction of IGF-1 in 2 of 8 patients in female patients with uncontrolled acromegaly [85]. However, an increase in tumor volume was observed in one patient with tumor expression of ER-*α* [85]. The side effects and selection of patients must be carefully evaluated in future studies.

#### 4.6.2. Peptide Receptor Radionuclide Therapy (PRRT)

Radio-labeled SSTR ligands are a form of target therapy for neuroendocrine tumors that belongs to peptide receptor radionuclide therapy (PRRT). The ligands bind to SSTRs and deliver radiation directly and specifically to tumors that express SSTRs. Therefore, the risk of systemic side effects is lower than conventional radiation therapy. The effects of PRRT on aggressive/refractory PitNETs have been tested in about 20 patients, and the efficacy is mild [86, 87]. Of the several cases reported to have aggressive/refractory GH-secreting PitNETs, one study showed decreased tumor growth and another exhibited stable disease for a follow-up of one year [88, 89]. Studies with a larger sample size are in demand for future research. In addition, upregulation of SSTRs with PRRT therapy and the application of radio-labeled SSTR antagonists might be alternative options for better outcomes [90–92].

#### 4.6.3. Inhibition on the VEGF and Tyrosine Kinase Pathways

Angiogenesis is a process of blood vessel growth that is significant for tumor progression and metastasis [93]. Activation of the vascular endothelial growth factor (VEGF) signaling pathway contributes to angiogenesis, and therefore, the development and pathological process in many solid tumors, including PitNETs. Invasive PitNETs have been shown to have higher vascular density and high VEGF expression than noninvasive PitNETs [94, 95]. The idea of anti-VEGF therapy in aggressive/refractory PitNETs and pituitary carcinomas has been tested in several case studies [96]. Anti-VEGF therapy includes the application of VEGF antibodies (Bevacizumab) and VEGF receptor antagonists, mainly known as tyrosine kinase inhibitors (TKIs, for example, sunitinib, sorafenib, and apatinib). In addition to anti-VEGF effects, TKIs target several other proteins at the same time, including the ErbB family signaling pathway, which may also contribute to the treatment of PitNETs [97]. Promising results were observed in 21 cases reported with VEGF and tyrosine kinase-targeted therapy [96]. Among them, two cases were diagnosed with aggressive/refractory GH-secreting PitNETs [98, 99]. A patient treated with bevacizumab was shown to have a partial response to radiological criteria and stable disease to biochemical criteria [99]. The other exhibited with stable disease in radiological criteria and partial response in biochemical criteria [98] after apatinib treatment. Side effects of VEGF and tyrosine kinase-targeted therapies mainly relate to the inhibition of the physiological role in the two on cardiovascular system, renal system, wound healing, hematologic system, etc. [100, 101].

#### 4.6.4. PI3K/Akt/mTOR Inhibitor

The phosphatidylinositol 3-kinase/mammalian target of rapamycin (PI3K/Akt/mTOR) pathway participates in the regulation of cell survival, growth, protein synthesis, and cellular metabolism [102]. Hyperactivation and/or upregulation of the mTOR signaling pathway are reported in neuroendocrine tumors, including PitNETs [103, 104]. Inhibiting PI3K/Akt/mTOR pathway could be a potential therapeutical option for PitNETs [105]. Jalali et al. reported that mTOR inhibition reduced tumor growth rate in pituitary adenomas and more importantly resulted in control of GH expression and IGF-1 secretion in a mouse model [106]. Everolimus (EVE) is an FDA-approved mTOR inhibitor for the treatment of neuroendocrine tumors. To our knowledge, only several cases of PitNETs have been treated with EVE, the effect is very subtle, and none of them are GH-secreting PitNETs [23, 107–109]. Side effects include stomatitis, rash, diarrhea, fatigue, etc., which is often mild and moderate. Some rare and severe adverse events with mTOR inhibitors have been reported, which could result in discontinuation of treatment and fatal outcome, for example, infection and pneumonitis [110]. Some *in vitro* and animal studies have shown the effects of inhibition of PI3K/Akt/mTOR pathway on reducing tumor cell viability and GH secretion in somatotroph PitNETs [111–113]. More preclinical and clinical studies are needed to validate the safety and efficacy of inhibition of PI3K/Akt/mTOR pathway on managing aggressive/refractory GH-secreting PitNETs.

#### 4.6.5. Immunotherapy

In recent years, immunotherapy with immune checkpoint inhibitors (ICIs) has been suggested for PitNETs, as the tumors have tumor-infiltrating T lymphocytes and express programmed death ligand 1 (PD-L1) [114–116]. Changes in tumor immune microenvironment and PD-L1 expression are observed in aggressive/refractory PitNETs, indicating checkpoint blockade immunotherapy for these cases [117]. In a recent study, Cossu et al. demonstrated that PD-L1 expression was associated with proliferative grades of Trouillas' classification and p53 expression [118]. They also confirmed a higher expression of PD-L1 in somatotroph tumors. Raverot et al. summarized in their review last year that there were only 10 cases of aggressive/refractory PitNETs (*n* = 2) and pituitary carcinomas (*n* = 8) treated with ICIs [15]. None of them are GH-secreting tumors. In the reported studies, five carcinomas presented with partial response and two carcinomas as stable disease. Side effects of ICIs include fever, hepatitis, asthenia, anorexia, hypophysitis, and other endocrine toxicities [119]. Two phase II studies are currently recruiting PitNET patients for immunotherapy with ICIs, nivolumab, and ipilimumab (NCT04042753 and NCT02834013). Hopefully, good efficacy and no serious adverse events will be reported in the future.

### 4.7. Follow-Up Strategies

Life-long follow-up is recommended for patients diagnosed with aggressive/refractory GH-secreting PitNETs [17] and ideally with MDT collaboration throughout. The clinical presentation, biochemical results, pituitary MRI, disease complications, and different treatment strategies must be evaluated in the follow-ups of GH-secreting PitNETs [41]. For aggressive/refractory cases, more frequent follow-up may be required. ESE suggested that imaging and complete endocrine evaluation be performed every 3–12 months depending on the prior tumor growth rate and/or tumor location (proximity to vital structures) and clinical context [17].

## 5. Conclusions

The new nomenclature system published in 2022 by WHO [9] provides the possibility that early use of more aggressive or novel therapies be considered for patients diagnosed with aggressive/refractory PitNETs including aggressive/refractory acromegaly/gigantism. Although there are some potential markers that indicate aggressiveness/invasiveness of GH-secreting PitNETs, no definitive conclusions have been made about how to diagnose aggressive/refractory GH-secreting PitNETs, which hinders further research to develop new treatment strategies. Officially proposed/published diagnostic criteria are urgently needed.

## Data Availability

No data were used to support this study.

## References

[1] Asa S. L., Casar-Borota O., Chanson P. (2017). From pituitary adenoma to pituitary neuroendocrine tumor (PitNET): an International Pituitary Pathology Club proposal. *Endocrine-Related Cancer*.

[2] Dekkers O. M., Karavitaki N., Pereira A. M. (2020). The epidemiology of aggressive pituitary tumors (and its challenges). *Reviews in Endocrine & Metabolic Disorders*.

[3] Asa S. L., Mete O., Perry A., Osamura R. Y. (2022). Overview of the 2022 WHO classification of pituitary tumors. *Endocrine Pathology*.

[4] Dai C., Kang J., Liu X., Yao Y., Wang H., Wang R. (2021). How to classify and define pituitary tumors: recent advances and current controversies. *Frontiers in Endocrinology*.

[5] Shimon I., Jallad R. S., Fleseriu M., Yedinak C. G., Greenman Y., Bronstein M. D. (2015). Giant GH-secreting pituitary adenomas: management of rare and aggressive pituitary tumors. *European Journal of Endocrinology*.

[6] Cuevas-Ramos D., Carmichael J. D., Cooper O. (2015). A structural and functional acromegaly classification. *Journal of Clinical Endocrinology and Metabolism*.

[7] Melmed S., Casanueva F. F., Klibanski A. (2013). A consensus on the diagnosis and treatment of acromegaly complications. *Pituitary*.

[8] Di I. A., Rotondo F., Syro L. V., Cusimano M. D., Kovacs K. (2014). Aggressive pituitary adenomas--diagnosis and emerging treatments. *Nature Reviews Endocrinology*.

[9] Liu X., Dai C., Feng M., Li M., Chen G., Wang R. (2021). Diagnosis and treatment of refractory pituitary adenomas: a narrative review. *Gland Surgery*.

[10] Mete O., Cintosun A., Pressman I., Asa S. L. (2018). Epidemiology and biomarker profile of pituitary adenohypophysial tumors. *Modern Pathology*.

[11] Asa S. L., Ezzat S. (2021). An update on pituitary neuroendocrine tumors leading to acromegaly and gigantism. *Journal of Clinical Medicine*.

[12] Mete O., Lopes M. B. (2017). Overview of the 2017 WHO classification of pituitary tumors. *Endocrine Pathology*.

[13] Hernandez-Ramirez L. C. (2020). Potential markers of disease behavior in acromegaly and gigantism. *Expert Review of Endocrinology and Metabolism*.

[14] Zhang S., Yao S., Chen J. (2023). Correlation between tumor invasion and somatostatin receptor subtypes in acromegaly. *Journal of Neurosurgery*.

[15] Raverot G., Ilie M. D., Lasolle H. (2021). Aggressive pituitary tumours and pituitary carcinomas. *Nature Reviews Endocrinology*.

[16] Popescu M. N., Ionescu E., Iovanescu L. C. (2013). Clinical aggression of prolactinomas: correlations with invasion and recurrence. *Romanian Journal of Morphology and Embryology*.

[17] Raverot G., Burman P., McCormack A. (2018). European Society of Endocrinology Clinical Practice Guidelines for the management of aggressive pituitary tumours and carcinomas. *European Journal of Endocrinology*.

[18] Dai C., Feng M., Liu X. (2016). Refractory pituitary adenoma: a novel classification for pituitary tumors. *Oncotarget*.

[19] Ezzat S., Gaspo R., Serri O., Ur E., Chik C. L. A. (2009). Canadian multi-centre, open-label long-term study of Pegvisomant treatment in refractory acromegaly. *Clinical an Investigative Medicine*.

[20] Huan C., Cui G., Lu C., Qu X., Han T. (2015). Role of Ki-67 in acromegalic patients with hyperprolactinemia: retrospective analysis in 61 Chinese Patients. *Pakistan journal of pharmaceutical sciences*.

[21] Burman P., Trouillas J., Losa M. (2022). Aggressive pituitary tumours and carcinomas, characteristics and management of 171 patients. *European Journal of Endocrinology*.

[22] Fernandez-Rodriguez E., Casanueva F. F., Bernabeu I. (2015). Update on prognostic factors in acromegaly: is a risk score possible?. *Pituitary*.

[23] McCormack A., Dekkers O. M., Petersenn S. (2018). Treatment of aggressive pituitary tumours and carcinomas: results of a European Society of Endocrinology (ESE) survey 2016. *European Journal of Endocrinology*.

[24] Caimari F., Korbonits M. (2016). Novel genetic causes of pituitary adenomas. *Clinical Cancer Research*.

[25] Kasuki L., Raverot G. (2020). Definition and diagnosis of aggressive pituitary tumors. *Reviews in Endocrine & Metabolic Disorders*.

[26] van der Lely A. J., Harris A. G., Lamberts S. W. (1992). The sensitivity of growth hormone secretion to medical treatment in acromegalic patients: influence of age and sex. *Clinical Endocrinology*.

[27] Ezzat S., Forster M. J., Berchtold P., Redelmeier D. A., Boerlin V., Acromegaly H. A. G. (1994). Clinical and biochemical features in 500 patients. *Medicine (Baltimore)*.

[28] Nomikos P., Buchfelder M., Fahlbusch R. (2005). The outcome of surgery in 668 patients with acromegaly using current criteria of biochemical cure. *European Journal of Endocrinology*.

[29] Ramírez-Rentería C., Hernández-Ramírez L. C. (2024). Genetic diagnosis in acromegaly and gigantism: from research to clinical practice. *Best Practice & Research Clinical Endocrinology & Metabolism*.

[30] Marques P., Caimari F., Hernandez-Ramirez L. C. (2020). Significant benefits of AIP testing and clinical screening in familial isolated and young-onset pituitary tumors. *Journal of Clinical Endocrinology and Metabolism*.

[31] Stratakis C. A., Tichomirowa M. A., Boikos S. (2010). The role of germline AIP, MEN1, PRKAR1A, CDKN1B and CDKN2C mutations in causing pituitary adenomas in a large cohort of children, adolescents, and patients with genetic syndromes. *Clinical Genetics*.

[32] Tichomirowa M. A., Barlier A., Daly A. F. (2011). High prevalence of AIP gene mutations following focused screening in young patients with sporadic pituitary macroadenomas. *European Journal of Endocrinology*.

[33] Beckers A., Aaltonen L. A., Daly A. F., Karhu A. (2013). Familial isolated pituitary adenomas (FIPA) and the pituitary adenoma predisposition due to mutations in the aryl hydrocarbon receptor interacting protein (AIP) gene. *Endocrine Reviews*.

[34] Das L., Gupta N., Dutta P. (2021). Early initiation of temozolomide therapy may improve response in aggressive pituitary adenomas. *Frontiers in Endocrinology*.

[35] Daly A. F., Tichomirowa M. A., Petrossians P. (2010). Clinical characteristics and therapeutic responses in patients with germ-line AIP mutations and pituitary adenomas: an international collaborative study. *Journal of Clinical Endocrinology and Metabolism*.

[36] Thakker R. V. (2014). Multiple endocrine neoplasia type 1 (MEN1) and type 4 (MEN4). *Mollecular and Cellular Endocrinology*.

[37] Verges B., Boureille F., Goudet P. (2002). Pituitary disease in MEN type 1 (MEN1): data from the France-Belgium MEN1 multicenter study. *Journal of Clinical Endocrinology and Metabolism*.

[38] Thapar K., Kovacs K., Stefaneanu L. (1997). Overexpression of the growth-hormone-releasing hormone gene in acromegaly-associated pituitary tumors. An event associated with neoplastic progression and aggressive behavior. *American Journal Of Pathology*.

[39] Yarman S., Kurtulmus N., Canbolat A., Bayindir C., Bilgic B., Ince N. (2010). Expression of Ki-67, p53 and vascular endothelial growth factor (VEGF) concomitantly in growth hormone-secreting pituitary adenomas; which one has a role in tumor behavior. *Neuroendocrinology Letters*.

[40] Katznelson L., Laws Jr. E. R., Melmed S. (2014). Acromegaly: an endocrine society clinical practice guideline. *Journal of Clinical Endocrinology and Metabolism*.

[41] Melmed S., Bronstein M. D., Chanson P. (2018). A consensus statement on acromegaly therapeutic outcomes. *Nature Reviews Endocrinology*.

[42] Fleseriu M., Biller B. M. K., Freda P. U. (2021). A pituitary society update to acromegaly management guidelines. *Pituitary*.

[43] Eisenhauer E. A., Therasse P., Bogaerts J. (2009). New response evaluation criteria in solid tumours: revised RECIST guideline (version 1.1). *European Journal of Cancer*.

[44] Donoho D. A., Bose N., Zada G., Carmichael J. D. (2017). Management of aggressive growth hormone secreting pituitary adenomas. *Pituitary*.

[45] Knosp E., Steiner E., Kitz K., Matula C. (1993). Pituitary adenomas with invasion of the cavernous sinus space: a magnetic resonance imaging classification compared with surgical findings. *Neurosurgery*.

[46] Starnoni D., Daniel R. T., Marino L., Pitteloud N., Levivier M., Messerer M. (2016). Surgical treatment of acromegaly according to the 2010 remission criteria: systematic review and meta-analysis. *Acta Neurochirurgica*.

[47] Bianchi A., Chiloiro S., Giampietro A. (2023). Multidisciplinary management of difficult/aggressive growth-hormone pituitary neuro-endocrine tumors. *Frontiers in Endocrinology*.

[48] Giustina A., Barkhoudarian G., Beckers A. (2020). Multidisciplinary management of acromegaly: a consensus. *Reviews in Endocrine & Metabolic Disorders*.

[49] Puig D. M. (2015). Treatment of acromegaly in the era of personalized and predictive medicine. *Clinical Endocrinology*.

[50] Kasuki L., Wildemberg L. E., Gadelha M. R. (2018). Management of endocrine disease: personalized medicine in the treatment of acromegaly. *European Journal of Endocrinology*.

[51] de Macêdo Filho L. J. M., Diógenes A. V. G., Barreto E. G. (2022). Endoscopic endonasal resection of the medial wall of the cavernous sinus and its impact on outcomes of pituitary surgery: a systematic review and meta-analysis. *Brain Sciences*.

[52] Mohyeldin A., Katznelson L. J., Hoffman A. R. (2022). Prospective intraoperative and histologic evaluation of cavernous sinus medial wall invasion by pituitary adenomas and its implications for acromegaly remission outcomes. *Scientific Reports*.

[53] Bakhsheshian J., Wheeler S., Strickland B. A. (2019). Surgical outcomes following repeat transsphenoidal surgery for nonfunctional pituitary adenomas: a retrospective comparative study. *Operative Neurosurgery (Hagerstown).*.

[54] Graillon T., Castinetti F., Fuentes S., Gras R., Brue T., Dufour H. (2020). Transcranial approach in giant pituitary adenomas: results and outcome in a modern series. *Journal of Neurosurgical Sciences*.

[55] Ershadinia N., Tritos N. A. (2022). Diagnosis and treatment of acromegaly: an update. *Mayo Clinical Procedings*.

[56] Coopmans E. C., van der Lely A. J., Neggers S. (2022). Approach to the patient with treatment-resistant acromegaly. *Journal of Clinical Endocrinology Metabolism*.

[57] Albarel F., Cuny T., Graillon T., Dufour H., Brue T., Castinetti F. (2022). Preoperative Medical treatment for patients with acromegaly: yes or no?. *Journal of Endocrine Society*.

[58] Melmed S. (2016). New therapeutic agents for acromegaly. *Nature Reviews Endocrinology*.

[59] Antunes X., Kasuki L., Gadelha M. R. (2021). New and emerging pharmacological treatment options for acromegaly. *Expert Opinion on Pharmacotherapy*.

[60] Del Porto L. A., Liubinas S. V., Kaye A. H. (2011). Treatment of persistent and recurrent acromegaly. *Journal of Clinical Neuroscience*.

[61] Li X., Li Y., Cao Y. (2017). Safety and efficacy of fractionated stereotactic radiotherapy and stereotactic radiosurgery for treatment of pituitary adenomas: a systematic review and meta-analysis. *Journal of Neurology Science.*.

[62] Abu D. A. M., Asi N., Farah W. H. (2015). Radiotherapy versus radiosurgery in treating patients with acromegaly: a systematic review and meta-analysis. *Endocrine Practice*.

[63] Marquez Y., Tuchman A., Zada G. (2012). Surgery and radiosurgery for acromegaly: a review of indications, operative techniques, outcomes, and complications. *International Journal of Endocrinology*.

[64] Lee C. C., Vance M. L., Lopes M. B., Xu Z., Chen C. J., Sheehan J. (2015). Stereotactic radiosurgery for acromegaly: outcomes by adenoma subtype. *Pituitary*.

[65] Knappe U. J., Petroff D., Quinkler M. (2020). Fractionated radiotherapy and radiosurgery in acromegaly: analysis of 352 patients from the German Acromegaly Registry. *European Journal of Endocrinology*.

[66] Rowe J., Grainger A., Walton L., Silcocks P., Radatz M., Kemeny A. (2007). Risk of malignancy after gamma knife stereotactic radiosurgery. *Neurosurgery*.

[67] Wilson P. J., De-Loyde K. J., Williams J. R., Smee R. I. (2013). Acromegaly: a single centre’s experience of stereotactic radiosurgery and radiotherapy for growth hormone secreting pituitary tumours with the linear accelerator. *Journal of Clinical Neuroscience*.

[68] Zheng Q., Huang Y., Lin W., Cai L., Wen J., Chen G. (2020). Comparing stereotactic radiosurgery and fractionated stereotactic radiotherapy in treating patients with growth hormone-secreting adenomas: a systematic review and meta-analysis. *Endocrine Practice*.

[69] Fadul C. E., Kominsky A. L., Meyer L. P. (2006). Long-term response of pituitary carcinoma to temozolomide. *Report of two cases. Journal of Neurosurgery*.

[70] Lim S., Shahinian H., Maya M. M., Yong W., Temozolomide H. A. P. (2006). A novel treatment for pituitary carcinoma. *Lancet Oncology*.

[71] Syro L. V., Uribe H., Penagos L. C. (2006). Antitumour effects of temozolomide in a man with a large, invasive prolactin-producing pituitary neoplasm. *Clinical Endocrinology*.

[72] Lasolle H., Cortet C., Castinetti F. (2017). Temozolomide treatment can improve overall survival in aggressive pituitary tumors and pituitary carcinomas. *European Journal of Endocrinology*.

[73] Elbelt U., Schlaffer S. M., Buchfelder M. (2020). Efficacy of temozolomide therapy in patients with aggressive pituitary adenomas and carcinomas-a German survey. *Journal of Clinical Endocrinology and Metabolism*.

[74] Luo M., Tan Y., Chen W. (2021). Clinical efficacy of temozolomide and its predictors in aggressive pituitary tumors and pituitary carcinomas: a systematic review and meta-analysis. *Frontiers in Neurology*.

[75] Kontogeorgos G., Thodou E., Osamura R. Y., Lloyd R. V. (2022). High-risk pituitary adenomas and strategies for predicting response to treatment. *Hormones*.

[76] Morin E., Berthelet F., Weisnagel J., Bidlingmaier M., Serri O. (2012). Failure of temozolomide and conventional doses of pegvisomant to attain biochemical control in a severe case of acromegaly. *Pituitary*.

[77] Ishida A., Shichi H., Fukuoka H., Shiramizu H., Inoshita N., Yamada S. (2022). Temozolomide and capecitabine treatment for an aggressive somatotroph pituitary tumor: a case report and literature review. *Frontiers in Oncology*.

[78] Wang X., Hu C., Li Y., Ren B., Yin G. (2022). Chemotherapy of capecitabine plus temozolomide for refractory pituitary adenoma after tumor resection and its impact on serum prolactin, IGF-1, and growth hormone. *Journal of Oncology*.

[79] Albright F., Reifenstein Jr. E. G. (1946). Effect of estrogens in acromegaly. *Transactions. Conference on Metabolic Aspects of Convalescence*.

[80] Shimon I., Barkan A. (2012). Estrogen treatment for acromegaly. *Pituitary*.

[81] Duarte F. H., Jallad R. S., Bronstein M. D. (2016). Estrogens and selective estrogen receptor modulators in acromegaly. *Endocrine*.

[82] Stone J. C., Clark J., Cuneo R., Russell A. W., Doi S. A. (2014). Estrogen and selective estrogen receptor modulators (SERMs) for the treatment of acromegaly: a meta-analysis of published observational studies. *Pituitary*.

[83] Dimaraki E. V., Symons K. V., Barkan A. L. (2004). Raloxifene decreases serum IGF-I in male patients with active acromegaly. *European Journal of Endocrinology*.

[84] Duarte F. H., Jallad R. S., Bronstein M. D. (2015). Clomiphene citrate for treatment of acromegaly not controlled by conventional therapies. *Journal of Clinical Endocrinology and Metabolism*.

[85] Magalhaes J., Ventura N., Lamback E. B., Da Silva D., Camacho A. H., Kasuki L. (2022). A prospective study on the efficacy of oral estrogen in female patients with acromegaly. *Pituitary*.

[86] Ilie M. D., Lasolle H., Raverot G. (2019). Emerging and novel rreatments for pituitary tumors. *Journal of Clinical Medicine*.

[87] Nakano-Tateno T., Lau K. J., Wang J. (2021). Multimodal non-surgical treatments of aggressive pituitary tumors. *Frontiers in Endocrinology*.

[88] Waligorska-Stachura J., Gut P., Sawicka-Gutaj N. (2016). Growth hormone-secreting macroadenoma of the pituitary gland successfully treated with the radiolabeled somatostatin analog (90)Y-DOTATATE: case report. *Journal of Neurosurgery*.

[89] Assadi M., Nemati R., Shooli H. (2020). An aggressive functioning pituitary adenoma treated with peptide receptor radionuclide therapy. *European Journal of Nuclear Medicine and Molecular Imaging*.

[90] Taelman V. F., Radojewski P., Marincek N. (2016). Upregulation of key molecules for targeted imaging and therapy. *Journal of Nuclear Medicine*.

[91] Nicolas G. P., Morgenstern A., Schottelius M., Fani M. (2018). New developments in peptide receptor radionuclide therapy. *Journal of Nuclear Medicine*.

[92] Brabander T., Nonnekens J., Hofland J. (2019). The next generation of peptide receptor radionuclide therapy. *Endocrine-Related Cancer*.

[93] Kerbel R. S. (2008). Tumor angiogenesis. *New England Journal of Medicine*.

[94] Lloyd R. V., Scheithauer B. W., Kuroki T., Vidal S., Kovacs K., Stefaneanu L. (1999). Vascular endothelial growth factor (VEGF) expression in human pituitary adenomas and carcinomas. *Endocrine Pathology*.

[95] Vidal S., Kovacs K., Horvath E., Scheithauer B. W., Kuroki T., Lloyd R. V. (2001). Microvessel density in pituitary adenomas and carcinomas. *Virchows Archivvirchows*.

[96] Dai C., Liang S., Sun B., Li Y., Kang J. (2021). Anti-VEGF therapy in refractory pituitary adenomas and pituitary carcinomas: a review. *Frontiers in Oncology*.

[97] Ben-Shlomo A., Cooper O. (2017). Role of tyrosine kinase inhibitors in the treatment of pituitary tumours: from bench to bedside. *Current Opinion in Endocrinology Diabetes and Obesity*.

[98] Wang Y., He Q., Meng X. (2019). YN968D1) and temozolomide in recurrent invasive pituitary adenoma: case report and literature review. *World Neurosurgery*.

[99] Dutta P., Reddy K. S., Rai A. (2019). Bevacizumab, radiotherapy, and Pegvisomant treatment of an AIP mutation‒positive child. *Journal of Clinical Endocrinology and Metabolism*.

[100] Chen H. X., Cleck J. N. (2009). Adverse effects of anticancer agents that target the VEGF pathway. *Nature Reviews Clinical Oncology*.

[101] Hartmann J. T., Haap M., Kopp H. G., Lipp H. P. (2009). Tyrosine kinase inhibitors-a review on pharmacology, metabolism and side effects. *Current Drug Metabolism*.

[102] Engelman J. A., Luo J., Cantley L. C. (2006). The evolution of phosphatidylinositol 3-kinases as regulators of growth and metabolism. *Nature Reviews Genetics*.

[103] Musat M., Korbonits M., Kola B. (2005). Enhanced protein kinase B/Akt signalling in pituitary tumours. *Endocrine-Related Cancer*.

[104] Sajjad E. A., Zielinski G., Maksymowicz M., Hutnik L., Bednarczuk T., Wlodarski P. (2013). mTOR is frequently active in GH-secreting pituitary adenomas without influencing their morphopathological features. *Endocrine Pathology*.

[105] Monsalves E., Juraschka K., Tateno T. (2014). The PI3K/AKT/mTOR pathway in the pathophysiology and treatment of pituitary adenomas. *Endocrine-Related Cancer*.

[106] Jalali S., Monsalves E., Tateno T., Zadeh G. (2016). Role of mTOR Inhibitors in growth hormone-producing pituitary adenomas harboring different FGFR4 genotypes. *Endocrinology*.

[107] Jouanneau E., Wierinckx A., Ducray F. (2012). New targeted therapies in pituitary carcinoma resistant to temozolomide. *Pituitary*.

[108] Donovan L. E., Arnal A. V., Wang S. H., Odia Y. (2016). Widely metastatic atypical pituitary adenoma with mTOR pathway STK11(F298L) mutation treated with everolimus therapy. *CNS Oncology*.

[109] Zhang D., Way J. S., Zhang X. (2019). Effect of Everolimus in treatment of aggressive prolactin-secreting pituitary adenomas. *Journal of Clinical Endocrinology and Metabolism*.

[110] Lee L., Ito T., Jensen R. T. (2018). Everolimus in the treatment of neuroendocrine tumors: efficacy, side-effects, resistance, and factors affecting its place in the treatment sequence. *Expert Opinion on Pharmacotherapy*.

[111] Di P. C., Gentilin E., Falletta S. (2018). PI3K/Akt/mTOR pathway involvement in regulating growth hormone secretion in a rat pituitary adenoma cell line. *Endocrine*.

[112] Ji C., Xu W., Ding H. (2022). The p300 inhibitor A-485 exerts antitumor activity in growth hormone pituitary adenoma. *Journal of Clinical Endocrinology and Metabolism*.

[113] Pivonello C., Patalano R., Solari D. (2018). Effect of combined treatment with a pan-PI3K inhibitor or an isoform-specific PI3K inhibitor and everolimus on cell proliferation in GH-secreting pituitary tumour in an experimental setting. *Endocrine*.

[114] Wang P. F., Wang T. J., Yang Y. K. (2018). The expression profile of PD-L1 and CD8(+) lymphocyte in pituitary adenomas indicating for immunotherapy. *Journal of Neurooncology*.

[115] Mei Y., Bi W. L., Greenwald N. F. (2016). Increased expression of programmed death ligand 1 (PD-L1) in human pituitary tumors. *Oncotarget*.

[116] Lu J. Q., Adam B., Jack A. S., Lam A., Broad R. W., Chik C. L. (2015). Immune cell infiltrates in pituitary adenomas: more macrophages in larger adenomas and more T cells in growth hormone adenomas. *Endocrine Pathology*.

[117] Dai C., Liang S., Sun B., Kang J. (2020). The progress of immunotherapy in refractory pituitary adenomas and pituitary carcinomas. *Frontiers in Endocrinology*.

[118] Cossu G., La Rosa S., Brouland J. P. (2023). PD-L1 expression in pituitary neuroendocrine tumors/pituitary adenomas. *Cancers*.

[119] Ramos-Casals M., Brahmer J. R., Callahan M. K. (2020). Immune-related adverse events of checkpoint inhibitors. *Nature Reviews Disease Primers*.

